# Impact of pharmacy worker training and deployment on access to essential medicines and health outcomes in Malawi: protocol for a cluster quasi-experimental evaluation

**DOI:** 10.1186/s13012-014-0156-2

**Published:** 2014-10-11

**Authors:** Solomon J Lubinga, Alisa M Jenny, Erin Larsen-Cooper, Jessica Crawford, Charles Matemba, Andy Stergachis, Joseph B Babigumira

**Affiliations:** Global Medicines Program, Department of Global Health, University of Washington, Harris Hydraulics Building, 1510 NE San Juan Road, Seattle, WA 98195-7965 USA; Pharmaceutical Outcomes Research and Policy Program, Department of Pharmacy, University of Washington, 1959 NE Pacific Street, Health Science Building, Seattle, WA 98195-7630 USA; VillageReach, 2900 Eastlake Ave. E., Seattle, WA 98102 USA; VillageReach, P.O. Box 31348, Lilongwe 3, Malawi; Department of Epidemiology, University of Washington, 1959 NE Pacific Street, Health Sciences Building, Seattle, WA 98195 USA

**Keywords:** Health workforce, Supply chain, Quasi-experiment, Impact evaluation, Malawi, Essential medicines, Malaria, Pneumonia, Diarrhea, Cost-effectiveness

## Abstract

**Background:**

Access to essential medicines is core to saving lives and improving health outcomes of people worldwide, particularly in the low- and middle-income countries. Having a trained pharmacy workforce to manage the supply chain and safely dispense medicines is critical to ensuring timely access to quality pharmaceuticals and improving child health outcomes.

**Methods/Design:**

This study measures the impact of an innovative pharmacy assistant training program in the low-income country of Malawi on access to medicines and health outcomes. We employ a cluster quasi-experimental design with pre-and post-samples and decision analytic modeling to examine access to and the use of medicines for malaria, pneumonia, and diarrhea for children less than 5 years of age. Two intervention districts, with newly trained and deployed pharmacy assistants, and two usual care comparison districts, matched on socio-economic, geographic, and health-care utilization indicators, were selected for the study. A baseline household survey was conducted in March 2014, prior to the deployment of pharmacy assistants to the intervention district health centers. Follow-up surveys are planned at 12- and 24-months post-deployment. In addition, interviews are planned with caregivers, and time-motion studies will be conducted with health-care providers at the health centers to estimate costs and resources use.

**Discussion:**

This impact evaluation is designed to provide data on the effects of a novel pharmacy assistant program on pharmaceutical systems performance, and morbidity and mortality for the most common causes of death for children under five. The results of this study should contribute to policy decisions about whether and how to scale up the health systems strengthening workforce development program to have the greatest impact on the supply chain and health outcomes in Malawi.

## Background

Prompt access to essential medicines can improve health outcomes, including saving lives in low- and middle-income countries (LMICs) [1,2]. However, access to most health-related commodities in LMICs remains low. For example, the mean public sector availability of essential medicines in the World Health Organization (WHO) African region is reported to be only 29.4% [3]. The lack of access to essential medicines has an especially large impact on children in Africa, potentially contributing to morbidity and mortality, from otherwise treatable diseases including malaria, pneumonia, and diarrhea [4]. In Malawi, UNICEF estimated that of 39,495 child deaths, 19% were due to malaria, 16% due to pneumonia, and 10% due to diarrheal diseases, a total of 45% [5].

Reasons for persistently low access to medicines include (1) inadequate financing; (2) regulatory problems; (3) lengthy procurement processes; (4) lack of incentives for maintaining sufficient stock levels; (5) poor logistics management (e.g., forecasting, distribution, information technology (IT) systems); (6) corruption; and (7) lack of qualified health workers to manage the medicines supply chain [2,6-8]. An effective medicines supply chain with sufficient numbers of well-trained and motivated human resources is an essential component of a robust health system [9,10]. However, according to the 2011 Malawi Health Sector Strategic Plan (HSSP) [11], there were only five pharmacists in the country’s public health system and only 24% of the established positions for pharmacy technicians were filled.

Task shifting has been proposed as a potential solution to increase the number of health workers in Malawi [12]. In their review of task-shifting studies, Mdege et al. [13] found that this may be an effective and cost-effective approach to expanding access to antiretroviral therapy where the health workforce is limited. Babigumira et al. [14] have also shown that task shifting may lead to cost and physician personnel savings in ART follow-up in Uganda and could contribute to mitigating health worker crises.

In the absence of qualified personnel, logistics and supply chain functions in pharmacies at health centers are performed by clinical health workers such as medical assistants and nursing aides [15,16]. This has the distinct disadvantage of diverting these cadres from their core duty of direct patient care [16]. And because they lack training in medicines logistics management, inefficiencies occur in supply chain management as well as clinical care [16]. Sometimes, pharmacy work is delegated to untrained hospital attendants or lay community members [12,15]; this can lead to adverse consequences for patients along with systems inefficiencies.

An alternative and potentially more efficient model is one in which pharmacy services, such as dispensing and logistics and supply management, at primary health centers are provided by a lower-level cadre of pharmacy worker called a pharmacy assistant (PA) [16,17]. This cadre of pharmacy worker receives up to 2 years of training in pharmacy culminating in the award of a Certificate in Pharmacy. Such a model is not new to Malawi. Until 1999, pharmacy assistants had been trained and deployed at district and central hospitals, but this program was discontinued as the Ministry of Health focused on developing a diploma and subsequently a degree program in Pharmacy [18,19]. Owing to the continued shortage of pharmacy workers at the health facility level [11,18], VillageReach, a Seattle-based international non-governmental organization with expertise in supply chain management, initiated an enhanced PA training program, in conjunction with the Malawi College of Health Sciences and the Malawi Ministry of Health, which is now being implemented in Malawi.

A crucial aspect of the program is that, as part of their training, PAs are deployed at district hospitals in their first year of training and in primary health centers in their second year of training for five months of intensive, supervised, field-based practicums. This has two potential advantages: 1) students should benefit from a supervised experiential learning environment that is characteristic of their future work environment, and 2) it should immediately alleviate the human resource gap at the primary health center and potentially, have an immediate effect on improving access to medicines at the community level.

There is a dearth of robust evidence on the impact of strengthening human resources capacity for medicines supply on access to medicines and health outcomes [15]. To bridge this knowledge gap, we designed an evaluation of the impact of PA training and deployment on access to essential medicines for malaria, pneumonia, and diarrhea (i.e., artemisinin-based combination therapies (ACTs), antibiotics, and oral rehydration salts (ORS), respectively), the leading causes of childhood mortality in Malawi [5]. In performing this evaluation, our hypothesis is that PA training and deployment will improve medicines management, logistics information flow for essential medicines, and supply chain functions at the primary health centers. This could lead to improved availability of medicines at primary health centers, increased access to essential medicines in the community, and improved health outcomes. We also hypothesize that PA training and deployment will be cost-effective from both the perspective of the Malawi Ministry of Health and a societal perspective. The specific objectives of this evaluation are the following:

1. To assess the impact of the PA training and deployment on operational efficiency at health centers;
2. To assess the impact of PA training and deployment on access to antimalarials, antibiotics, and ORS at the community level in children with fever, symptoms of pneumonia, and diarrhea, respectively;
3. To model the impact of PA training and deployment on mortality and disability-adjusted life-years (DALYs);
4. To estimate the costs and cost-effectiveness of PA training and deployment.

## Methods

### Study design

**Figure 1:**
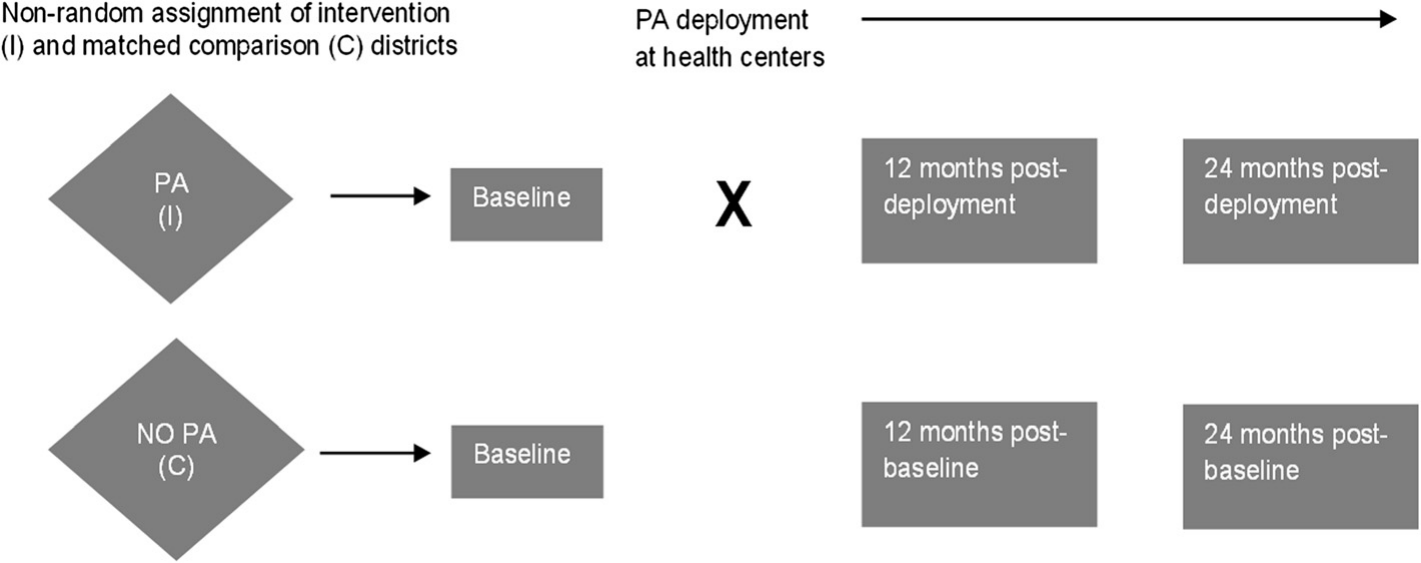
**Quasi-experimental study design, with 3 separate cross-sectional samples.** One pre-intervention and two post-intervention samples at 12 and 24 months are conducted for the population-based household survey and the health center-based time-motion and patient surveys.

The evaluation includes three complementary components: (1) a health center (HC)-based time-motion and patient survey, (2) a population-based household survey, and (3) a model-based health and economic analysis. The HC-based time-motion and patient surveys as well as the population-based household survey use a quasi-experimental, separate pre-post samples design (Figure 1) [20]. The model-based health and economic analysis uses decision analytic methods.

The primary outcome of the time-motion and patient survey is operational efficiency, measured as patient wait time at HCs. The primary outcome of the household survey is access to ACTs, antibiotics, and ORS. We adopted the access-to-medicines (coverage rate) definition used in the Malawi Demographic and Health Survey (DHS), 2010 [21], i.e., the percentage of children with non-severe malaria, pneumonia, or diarrhea in the last 2 weeks who obtained a full course of ACT, antibiotics, or ORS, respectively. The primary outcomes of the model-based health and economic analysis are life-years (LY) gained, DALYs averted and incremental costs of the program.

### Study setting

**Figure 2:**
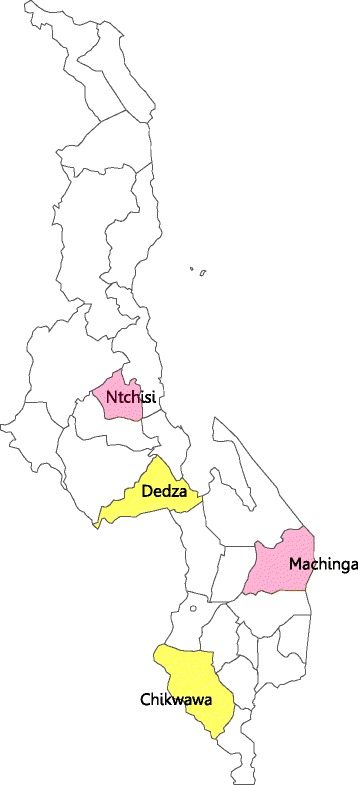
**Map of Malawi indicating study districts.** Ntchisi and Machinga marked in pink are the intervention sites and Chikwawa and Dedza marked in yellow are the comparison sites.

Malawi has 28 districts across three regions—North, Central, and South (Figure 2). Administratively, the districts are subdivided into traditional authorities (TAs). Each TA is composed of villages, which are the smallest administrative units. There are three levels of health care: (1) the primary level, which operates at the village level, consists of village clinics operating community-based Integrated Management of Childhood Illness (cIMCI), health posts, dispensaries, maternities, health centers, and community and rural hospitals; (2) the secondary level, which operates at the district level, consists of district hospitals; and (3) the tertiary level which consists of four specialist referral central hospitals [11]. The PAs are deployed to health centers, one of the seven different types of health facilities at the primary level.

### Intervention

The intervention is the 2-year certificate program to train and deploy a new cadre of PAs established in 2012. A partnership between the Malawi College of Health Sciences (MCHS), Malawi Ministry of Health (MoH), and the VillageReach, with technical assistance provided by the University of Washington, the program seeks to train and deploy at least 150 PAs to improve pharmaceutical management in rural HCs in Malawi. The intervention is being implemented in 18 districts selected from the three regions of the country where a motivated and knowledgeable pharmacy technician at the district hospital indicated a willingness to provide mentorship and technical support to PA trainees during their training at hospitals and at the HC.

During the first year of training, students undergo 10 weeks of class-based instruction at MCHS. At the end of this time period, half the students are deployed to district hospitals for a 5-month period of field training supervised by a pharmacy technician, and the other half of the students remain at the MCHS for additional didactic training; the students then change places for another 5 months. In the second year, half of the trainees are deployed to HCs in the intervention districts for 5 months while the other half will remain at the MCHS for class-based instruction; the students then change places for another 5 months. Therefore, during the second year, HCs in intervention districts have a trainee PA on-site for 10 of 12 months. Members of the teaching staff from MCHS conduct routine supervisory visits at both district hospitals and HCs to provide on-site mentorship and assess student progress.

At the HCs, PA trainees are responsible for health commodity management including stock room management, storage, stock cards and record keeping, placing emergency orders and collecting commodities from the district health office, and completing logistics management information system (LMIS) reports guided by the Malawi health commodities Logistics Management System Standard Operating Procedures manual [22]. Additionally, they are responsible for dispensing and providing counseling to patients and supervising and managing commodities used by the community health workers based out of their HC.

### Study sites

Four districts were purposively selected for the impact evaluation: Ntchisi and Machinga as the intervention sites and Chikwawa and Dedza as the comparison sites (Figure 2). These districts were matched on region; level of malaria, pneumonia, and diarrheal risk; access to the basic services (roads, water, education, health centers, and markets); geographic distinctions such as mountain vs. lake regions; and socioeconomic status of residents. The time-motion and patient surveys were conducted at four health centers, one from each district, as follows: Khuwi in Ntchisi district, Ntaja in Machinga district, Dolo in Chikwawa district, and Lobi HC in Dedza district. The household surveys were conducted in villages surrounding the four HCs chosen for the evaluation.

### Study procedures and sampling

Baseline surveys were conducted in March 2014 prior to PA deployment to HCs. Additional surveys are planned at 12 and 24 months after PA trainee deployment to the intervention sites. The post-intervention surveys are timed to occur after a PA trainee has been consistently available at the HC for at least 6 months. We assume that over this period, the trainee will have had sufficient time to settle into a working routine and will have had an impact on routine functioning of the logistics system and dispensing practices, and hence an impact on availability and use of medicines at the HC.

At each health center, time-motion and patient surveys will be conducted on each day of the work week from Monday to Friday for one full week. This is necessary to capture the day-to-day distribution of patient load. The survey samples consist of all consenting out-patients visiting the HC on the day of the survey. This is important to identify bottlenecks in service delivery. Research staff will provide each patient with the time-motion questionnaire that they will move with throughout the clinic visit. Health workers that interface with patients will fill out time sheets noting the time as soon as a patient appears at their station and the time when they leave their station. The health worker at the last health-care station will send the patient back to research staff for the patient survey. The research staff will also record the start and end times of the experiential survey.

The population-based household surveys employ a two-stage cluster sampling design. The study samples consist of randomly selected households with children between 2–59 months of age within census enumeration areas (EAs) adjacent to the each HC. Inclusion criteria are as follows: 1) household representative consent to participate in the study, and 2) at least one child between 2–59 months with reported symptoms of non-severe malaria, pneumonia, or diarrhea in the previous 2 weeks. For malaria, only those children for whom the caretaker or household representative reports a history of fever will be included. For children with non-severe pneumonia, those with a history of cough or difficulty breathing and a rapid respiratory rate will be included; for diarrhea, those who report a history of loose watery stools will be included [23]. These are children who should have received either ACTs for non-severe malaria, an antibiotic for non-severe pneumonia, or ORS for diarrhea. We will exclude all those with severe malaria or pneumonia and those under 2 months of age because ACTs are contraindicated in this age group, and any child under 2 months of age with cough or difficulty breathing is considered to have severe pneumonia. We will also exclude those children whose caregivers report them as having bloody stools.

In the first stage, we will identify all EAs within a district based on the data available from the Malawi National Statistics Office. Clusters will be selected based on their proximity to selected health centers in both the intervention and comparison districts. We will sample from the 30 closest EAs to each of the four health centers. In the second stage, we shall identify one or more central points in the village such as a market or school, choose a direction or route at random, count the number of households between that point and the edge of the village, and then select one of these as the starting point. Every subsequent tenth household will then be selected systematically until the targeted number of households for that cluster has been screened. Emphasis will be placed on ensuring as widespread coverage as possible in each EA and achieving a random or near-random selection of households. Procedures used in household sampling in each EA will be reported in detail. Households will be approached for screening according to the inclusion and exclusion criteria. For households with eligible and consenting participants, the survey questionnaire will be administered for each child who meets our inclusion criteria.

### Sample size

According to the Malawi DHS, 2010, approximately 35%, 7%, and 18% of children between 2–59 months reported having malaria, pneumonia symptoms, or a diarrhea episode, respectively, in the 2 weeks preceding the survey [21]. Of these children, 36%, 24%, and 69% received an ACT for malaria, antibiotic for pneumonia, and ORS for diarrhea, respectively [21]. Our aim is to detect modest and meaningful increases in access to ACTs, antibiotics, and ORS to 50%, 40%, and 80%, respectively, with 80% power and 5% precision. We would require a random sample of at least 390 children with non-severe malaria, 264 children with pneumonia, and 490 with diarrhea at each cross-section, divided equally between intervention and comparison groups to demonstrate the proposed effect sizes [24]. However, this is a population-based household cluster sample requiring initial screening of households for children reporting non-severe malaria, pneumonia, or diarrhea in the previous 2 weeks. Therefore, to achieve these sample sizes, given an expected prevalence of a febrile episode, pneumonia symptoms and diarrhea episode of 35%, 7%, and 18%, respectively, and assuming there are 1.5 children per household between 2–59 months old, a household non-response rate of 10%, and a design effect of 1.5 to account for within-cluster homogeneity in the primary outcome [21,25], we estimated that we needed to screen approximately 1,226 households for children with malaria, approximately 4,149 households for children with pneumonia, and 782 households for children with diarrhea. We chose the largest sample size, i.e., 4,150 households at each cross-section: 2,075 households in the intervention and 2,075 in the comparison districts.

### Study questionnaires

The HC-based time-motion and patient survey questionnaires were developed for this evaluation. The time-motion survey consists of a time-sheet for recording movements throughout the clinic, including the time of presentation to the health center, waiting times, and time-in and time-out for different services such as seeing the triage nursing assistant, the medical assistant, and/or the pharmacy worker. The patient survey consists of a series of questions related to the patient’s experience at the health center, any medicines prescribed by the clinician, and dispensed at the pharmacy.

The household survey questionnaire was adapted from the 2010 Malawi Demographic and Health Survey Household and Women’s questionnaires [21]. It consists of three sections: screening, household, and care-seeking and medicine-use sections. The screening section identifies potential participants who report having had symptoms suggestive of non-severe malaria, pneumonia, or diarrhea in the previous 2 weeks. The household section captures information on the demographic characteristics of all usual members of the household including their ages, gender, education, relationship to the household head, and marital status (of those above age 18). It also captures various socioeconomic indicators including household income and expenditures, access to safe water, electricity, and toilet facilities, type of fuel used to cook, ownership of material items like radios, televisions and cell phones, material of floors, roof and walls in the house, ownership of land, livestock and poultry, and ownership of a bank account. The care-seeking and medicine-use section first captures data on whether care was sought for the illness episode, and if care was sought, where, after how long, distance travelled, time and cost of seeking care. It also captures information on antimalarials, antibiotics, ORS, and other supportive treatments received, and the time and costs spent looking after the sick child.

The study questionnaires were translated to Chichewa, the other official language of Malawi (in addition to English), back-translated to ensure no loss of information, and edited further where necessary. We conducted a 1-week training of research assistants and supervisors at which we discussed the study procedures and performed an in-depth review of the questionnaires. The questionnaires were pretested, and minor changes made to both the questionnaires and study procedures.

### Analytic strategy

Data from the questionnaires will be double-entered and cross-validated in the Census and Survey Processing System, CSPro (US Census Bureau, USA). Analyses will be conducted using STATA version 12.1 (StataCorp LP, College Station, TX, USA). The decision analytic model for the economic evaluation will be analyzed in Microsoft Excel 2010 (Microsoft Corp., Redmond, WA, USA).

A socioeconomic status index will be constructed by principal component analysis of data on ownership of durable goods and housing characteristics collected in the household module of the survey questionnaire [26]. In bivariate analyses, we will summarize the characteristics of enrolled participants and households by intervention and comparison district as proportions for categorical variables and means (standard deviation) for continuous variables. Pearson’s chi-square tests will be used to compare proportions and *t*-tests to compare continuous variables between intervention and comparison groups.

In the primary analysis, we will use a difference-in-differences estimator within a multivariable regression framework [27,28], to estimate the causal effect of PA training and deployment on access to the tracer commodities, adjusting for potential confounders including 1) primary caregiver demographics—age, gender, education level, employment status, and marital status; 2) patient demographics—age and gender of the patient; 3) household characteristics—household income and expenditure, socioeconomic index group; and 4) proxies for access to care—distance (and time cost) to nearest health facility.

### Economic evaluation

We are developing a decision analytic model to represent the consequences of differential access to ACTs, antibiotics, and ORS on morbidity, mortality, DALYs, and costs due to malaria, pneumonia, and diarrhea, respectively. The modeling framework will take the form of a decision tree that tracks access to medicines. The morbidity consequence of access/no access to treatment is potential progression to severe disease or sequelae. The model will track care-seeking patterns and treatment patterns for severe disease or sequelae as well as survival. The probabilities of accessing an ACT, an antibiotic, or ORS will be derived from our quasi-experimental evaluation. We are systematically synthesizing current evidence from the literature to obtain all other model probabilities, life expectancies, and disability weights.

The economic evaluation is being conducted both from the perspective of the Malawi Government Ministry of Health and from a societal perspective. We shall estimate the direct and indirect costs of routine health service provision and program implementation in the intervention facilities and routine health service provision in the comparison facilities. These resource use and cost data shall be derived from the program and health center accounting systems. Using a time-motion study and patient interviews, we shall estimate the direct medical and non-medical costs and indirect costs (productivity losses) associated with treating malaria, pneumonia, and diarrhea in hospitals. All costs are being converted to 2014 US dollars (US$). Costs and outcomes will be discounted at 3% [29].

Incremental cost-effectiveness ratios (ICERs) will be calculated as the incremental cost per life-year saved and incremental cost per DALY averted. Scenario analyses and univariate and probabilistic sensitivity analyses will be performed to examine the impact of assumptions and uncertainty around model parameters on life-years gained, DALYs averted, and incremental costs.

### Ethical considerations

Ethical clearance was obtained from the University of Washington Institutional Review Board and the Malawi National Health Sciences Research Committee (NHSRC). Informed consent is being sought from all participants prior to administering the study instruments. The consent forms and study instruments are available in Chichewa and English.

### Study status

Baseline surveys, including the population-based household and HC-based time-motion and patient surveys, were conducted in March 2014. Implementation of the intervention began in April 2014. PA trainees have been deployed at the study health centers for 5 months.

## Discussion

This paper describes the protocol for a cluster quasi-experimental design study whose aim is to determine the impact of having trained pharmacy personnel in rural health centers on access to medicines and clinical care at health facilities. We are using decision analytic methods to model the impact of the intervention on health outcomes, specifically child morbidity and mortality and costs. This decision model will also form the basis of an assessment of the cost-effectiveness of the intervention.

### Innovation and potential impact

The lack of trained pharmacy staff at the health center service delivery level to manage medicines and supply chain has become a critical bottleneck in national efforts to improve access to medicines and primary health-care service delivery. The demand for pharmaceutical services, particularly with the advent of the HIV/AIDS epidemic, has increased markedly and points to the need for a comprehensive and sustainable scale up of the pharmaceutical workforce in low-income countries like Malawi. This project seeks to evaluate the causal effect of a potentially efficient, community-level intervention in which pharmaceutical services at lower-level health facilities are delegated to lower-level cadre pharmacy worker. We theorize that addressing this human resources constraint will improve medicines management, logistics information flow, and supply chain function at the health facility level, leading to improved medicines availability at public health facilities, access to essential medicines in the community and health outcomes. Although similar programs are already being implemented in Namibia, South Africa, and Tanzania, associated costs and impact on medicines availability at public health facilities have not been reported. Moreover, the impact of supply chain enhancements on health outcomes has also not been rigorously evaluated.

### Limitations

We limit our primary analysis to evaluating the impact of the intervention on community level access to the tracer medicines and the impact on service provision at the health facilities. Decision analytic methods (informed by the literature and our primary study) are used to extrapolate to health outcomes. As such, we are unable to directly quantify the direct impact of this program on actual morbidity and mortality. It was not possible to randomly assign the PAs to intervention and comparison districts, due to political and geographic constraints. It is therefore possible that there will be observed and unobserved differences in the baseline measures of the intervention and comparison groups that may account for differences in outcomes. However, the use of difference-in-differences analytic methods in a regression framework will allow us to account for both observed and unobserved confounders. There will likely remain questions of how much of the improvement is on account of training and deployment per se, and how much of it is due to better motivation of the newly deployed PAs. This will be impossible to tease out quantitatively.

As previously mentioned, differences in drug availability, resulting from national supply shortages, may present a challenge to the internal validity of the evaluation. However, we do not expect this would be differential between intervention and control districts. To protect against differential access resulting from differential availability of medicines at the district health offices, we selected districts that had a track record of maintaining adequate stocks of the tracer commodities throughout the previous year. Our assumption is that national shortages of medicines will affect facilities in both intervention and control districts similarly.

It is worthwhile to note that the impact of the PA program may extend beyond availability and consequently community access to the tracer commodities. Access to all products that are routinely used at the health centers may improve and as a consequence morbidity and mortality from the conditions that those commodities target, e.g., vaccines and vaccine preventable diseases. Therefore, any estimates we obtain may underestimate the potential impact of the program on overall under-5 mortality.

## Conclusions

This protocol describes the evaluation of an innovative human resources intervention aimed at improving the medicines supply chain in a low-income country, Malawi. It will generate robust evidence on the effect of such interventions on community-level access to essential medicines and the impact on clinic level operations. To further guide national- and region-wide policy implementation, we will model the impact of the intervention on costs, health outcomes, and cost-effectiveness.
